# Equine hepatocytes: isolation, cryopreservation, and applications to in vitro drug metabolism studies

**DOI:** 10.1002/prp2.268

**Published:** 2016-09-30

**Authors:** Khaled A. Shibany, Sabine Tötemeyer, Stefanie L. Pratt, Stuart W. Paine

**Affiliations:** ^1^ School of Veterinary Medicine and SciencesUniversity of NottinghamCollege RoadSutton BoningtonLeicestershireLE12 5RDUnited Kingdom

**Keywords:** Cell viability, cryopreservation, drug metabolism, equine, hepatocyte isolation

## Abstract

Despite reports of the successful isolation of primary equine hepatocytes, there are no published data regarding the successful cryopreservation of these isolated cells. In this study, a detailed description of the procedures for isolation, cryopreservation, and recovery of equine hepatocytes are presented. Furthermore, the intrinsic clearance (Cl_int_) and production of metabolites for three drugs were compared between freshly isolated and recovered cryopreserved hepatocytes. Primary equine hepatocytes were isolated using a two‐step collagenase perfusion method, with an average cell yield of 2.47 ± 2.62 × 10^6^ cells/g of perfused liver tissue and viability of 84.1 ± 2.62%. These cells were cryopreserved with William's medium E containing 10% fetal bovine serum with 10% DMSO. The viability of recovered cells, after a 30% Percoll gradient, was 77 ± 11% and estimated recovery rate was approximately 27%. These purified cells were used to determine the in vitro Cl_int_ of three drugs used in equine medicine; omeprazole, flunixin, and phenylbutazone, via the substrate depletion method. Cryopreserved suspensions gave a comparable estimation of Cl_int_ compared to fresh cells for these three drugs as well as producing the same metabolites. This work paves the way for establishing a bank of cryopreserved equine hepatocytes that can be used for estimating pharmacokinetic parameters such as the hepatic metabolic in vivo clearance of a drug as well as producing horse‐specific drug metabolites.

**Abbreviations**
BSA: bovine serum albumin
CLint: intrinsic clearance
DMSO: dimethyl sulfoxide
EGTA: ethylene glycol tetraacetic acid
EPC: effective plasma drug concentration
ESI: electrospray ionization
FBS: fetal bovine serum
HBSS: Hanks balanced salt solution
HPLC: high‐performance liquid chromatography
IPC: irrelevant plasma concentration
MS: mass spectrometer
PD: pharmacodynamics
PK: pharmacokinetics
TB: Trypan Blue
WEM: Williams medium E

## Introduction

Horses have both a commercial and a domestic role within society, ranging from companion animals to the equine sports and food industries. In all cases, the welfare of the horses must be considered, including appropriate medication when necessary. The optimal dosing regimen for a medication is dependent on a thorough understanding of the drug pharmacokinetics (PK). The clearance of a drug is particularly important, especially when determining the average concentration of a drug once steady‐state has been reached.

The equine sports industry is a multi‐billion dollar industry (British Horseracing Authority, 2013) and, while medication is allowed when the horse is in training, most international equine sports jurisdictions’ require the horse to be free of medication on the day of the competition. Therefore, where a horse competes having received medication in its training program, the term “free of any medication” has to be defined. One such definition can be a drug concentration in plasma that is less than the concentration required for a significant therapeutic effect. Toutain and Lassourd (2002) proposed an approach based upon the above definition that also takes into account variation in both PK and pharmacodynamic (PD) parameters for a population of horses. An estimate of the irrelevant plasma concentration (IPC) is based upon a pharmacologically effective plasma drug concentration (EPC) divided by an appropriate safety factor. The EPC is the average concentration at steady‐state and is estimated from the therapeutic dose, dosing interval, and clearance of the drug. The IPC can be used for deriving a drug screening limit, which in turn can be used in conjunction with drug in vivo PK profiles to obtain appropriate drug withdrawal times.

In vivo PK studies including bioanalysis are expensive as well as involving invasive sampling from the animal. It is now standard practice within the pharmaceutical industry to use in vitro tissue models to estimate the clearance of a drug in human and reduce the number of preclinical animals used. The use of primary hepatocytes has been practiced for more than five decades (Berry and Edwards 2001). Primary hepatocytes are considered as a “gold standard” for evaluating hepatic metabolism and toxicity of drugs and other xenobiotics in vitro and potential drug‐drug interactions (Dambach et al. 2005; Hewitt et al. 2007; Li 2007). Although freshly isolated hepatocytes have a limited viability in suspension of up to 4 h, this period is long enough to determine the metabolic stability of a drug and to allow identification of major metabolites (Jouin et al. 2006; Soldatow et al. 2013). The limited availability of fresh cells restrains their use, however, cryopreservation offers an option for long‐term storage of hepatocytes providing a continuous and sufficient “off the shelf” supply (Bachmann et al. 2003; Griffin and Houston 2004; Terry et al. 2010).

Fresh equine primary hepatocytes have successfully been isolated and cultured (Bakala et al. 2003; Stefanski et al. 2013). However, there is no literature evidence describing the cryopreservation and recovery of equine hepatocytes and the resulting cell viability and activity of xenobiotic metabolizing enzymes. A better understanding of equine drug metabolism is important for optimal administration regimens of drugs and the resulting effects of the applied treatment. As a consequence, equine veterinary surgeons will be better informed with regard to the outcomes of administered drugs and as a result potential drug‐drug interactions may be avoided. This will ultimately lead to improved welfare of the horse population. Furthermore, a greater understanding of how drugs are metabolized in the horse, including rates of metabolism and metabolites generated using in vitro systems, will aid the equine sports industry regulate for the abuse of medications and doping substances with a reduction in animal usage and overall costs.

The aim of this work was to develop and optimize a protocol for the cryopreservation of equine hepatocytes that can be recovered with good cell viability and metabolic activity for use in metabolism studies of the commonly used equine drugs omeprazole, flunixin, and phenylbutazone.

## Materials and Methods

### Materials

Collagenase and Calcium‐free Hanks Balanced Salt Solution (HBSS) ×10, HEPES buffer, William's medium E, and fetal bovine serum (FBS) were purchased from Thermo Fisher Scientific (Paisley, UK). Dimethyl sulfoxide, Percoll, flunixin, ketoprofen, phenylbutazone, diclofenac, and trypan blue were purchased from Sigma (Dorset, UK). Omeprazole and lanzaprozole were purchased from Tocris Bioscience (Bristol, UK). All other chemicals were of reagent grade.

### Liver samples

Livers were obtained from seven horses post slaughter (2–22 years old, of both sexes). The horses were slaughtered in accordance with the Welfare of Animals at the Time of Killing (UK) Regulations 2015. Liver lobes (preferably the left lateral, caudal, or quadrats segment) were excised by a single transverse cut and immediately after excising immersed in HBSS (0–2°C) prior to being transported to the laboratory.

### Hepatocyte preparation

For hepatocytes preparation, a “two‐step” perfusion procedure (chelating and collagenase perfusion) was used based upon Selgens’ method (Selgen 1976). Once in the laboratory, part of the liver lobe was excised to obtain on the cutting surface no more than two big hepatic vessels (5–8 mm in diameter). The resulting lobe segment (90–110 g) was cannulated and initially perfused with calcium‐free Hanks‐HEPES solution (44 mL/min/cannula) until all blood was removed and an outlet temperature of 37°C achieved (8–12 min). The liver segment was then perfused with Ca^2+^‐free HBSS supplemented with 0.1 mmol/L EGTA (Chelating step) for 10 min, without recirculation of the perfusate. This was followed by HBSS buffer (0.5 L) containing 5 mmol/L calcium chloride followed immediately by recirculating HBSS buffer containing 5 mmol/L calcium chloride and collagenase (0.1% w/v), through the liver segment for 15–25 min at the same flow rate. All perfusion buffers were prewarmed to 40°C with the exception of the collagenase solution which was prewarmed to 37°C. The digested liver segment was then transferred to a 10 cm Petri dish and disrupted gently, using pointed scissors to tear the liver capsule.

### Purification of the initial cell suspension

The digested liver tissue was dispersed in 100 mL HBSS buffer containing 0.1% w/v albumin at 4°C. The resulting cell suspension was twice filtered, first through a 250 *μ*m nylon mesh followed by a 100 *μ*m nylon mesh. Next, HBSS buffer containing albumin was added to the filtered suspension to reach a final volume of 300 mL. Once resuspended, the cell suspension was centrifuged for 5 min at 75*g* followed by 3 min at 50*g*. Cell pellets were resuspended in 45 mL HBSS buffer containing albumin and placed on ice.

### Determination of hepatocytes yield and viability

The yield and viability of freshly isolated hepatocytes were assessed with Trypan Blue (TB) dye exclusion test (Tennant 1964). A quantity of 15 *μ*L TB was mixed with the same volume of cell suspension and stained (nonviable) and unstained (viable) cells were counted in a hemocytometer. Viability % was determined from the percentage of live cells to total cell count.

### Cryopreservation of freshly isolated hepatocytes

Hepatocytes were cryopreserved according to a modified protocol by Adams et al. (1995): The main modification was use of 50% v/v Williams E medium, 40% v/v Fetal calf serum, and 10% v/v DMSO as cryopreservation media. Cells were pelleted at 50*g* for 3 min at 4°C, the supernatant was removed by aspiration, and the pellet was resuspended in cryopreservation media prepared fresh at the time of cryopreservation under sterile conditions. The cryopreservation medium was added dropwise while the suspension was gently shaken. After resuspension, the cells were placed on ice and aliquoted (1 mL of cell suspension/cryovial at a concentration of 1 × 10^7^ cells/mL). The vials were then transferred into Mr Frosty^®^ isopropanol Containers (Fisher, UK) and cooled at a rate of −1°C/min in a −80°C Freezer. After 18 h, the vials were transferred to liquid nitrogen for long‐term storage at −196°C.

### Cell recovery

Cryopreserved equine hepatocytes were recovered by thawing the vials in a 37°C water bath under gentle agitation. The cells were diluted 1:10 by drop wise addition of prewarmed WEM plus 10% fetal bovine serum as previously described by Terry et al. 2010. Cells were pelleted at 50*g* for 5 min at room temperature and suspended in prewarmed WEM. Hepatocyte yield and viability was determined immediately post‐thaw by TB exclusion.

### Recovery of cryopreserved hepatocytes for drug clearance assays

After thawing, viable cells were purified using two different Percoll gradients (25 and 30%). A quantity of 15 mL of the Percoll solution was overlaid with the content of one vial and centrifuged at 110*g* at 4°C for 20 min (Kreamer et al. 1986; SciKon Innovation 2010). After centrifugation, the dead cells and the cell debris were located at the interface while viable cells were pelleted. The pelleted cells were resuspended in WEM and washed by centrifugation for 3 min at 50*g*.

### Determination of intrinsic clearance

Freshly isolated or recovered cryopreserved hepatocytes were diluted to 1 million cells/mL, using WEM with 0.125% v/v bovine serum albumin (BSA), and were preincubated for 30 min at 37°C with 5% CO_2_ as previously described by Jouin et al. (2006). All incubations were performed in 1.2 mL cluster tubes (Sigma, Dorset, UK) in a 96‐well heater block (BioShake iQ), set at 750 rpm and 37°C with a total incubation volume of 1 mL. Drug stocks were prepared in DMSO at 100× final incubation concentration (5 *μ*mol/L). The reaction was initiated by adding 10 *μ*L of each drug stock to cell suspension. Then, 100 *μ*L aliquots were removed at 0, 5, 10, 20, and 30 min and quenched in 200 *μ*L of ice‐cold methanol containing internal standard (10 *μ*mol/L). Samples were subsequently frozen at −20°C until further analysis. Samples were centrifuged at 31*g* for 10 min at 4°C prior to HPLC analysis. The supernatant was removed and transferred into HPLC vials and analyzed.

### LC/MS analysis

Peak area ratios of omeprazole, flunixin, and phenylbutazone to their respective internal standard (lansoprazole, ketoprofen, and diclofenac) were determined using high‐performance liquid chromatography (Agilent 1100 HPLC Palo Alto, CA) and mass spectrometer detector (Quattro ultima VB250) HPLC/MS system. Separation was performed on a C18 column (Discovery, 5 *μ*m particle size, 15 cm × 3.0 mm) (Sigma, UK). The mobile phase consisted of Solvent A (10% methanol, 90% water, and 0.02% formic acid) and Solvent B (100% methanol containing 0.02% formic acid). The solvent flow rate was kept at a constant 0.5 mL/min with an upper pressure limit of 400 bar. All compounds were detected by positive electrospray ionization (ESI) in the selected ion monitoring mode using predetermined parent MH/Z and oxidative metabolites MH+16/Z. The intrinsic clearance was determined using the drug substrate depletion approach where the slope of the logarithm of MS parent drug response versus time was determined and converted into appropriate units.

### Statistical analysis

The data are presented as mean of seven cell preparations ±standard deviations. Statistical significance was estimated by the paired, two‐tailed Student's test. A *P* < 0.05 was considered significant.

## Results and Discussion

### Yield and viability of fresh and cryopreserved equine hepatocytes

The average of total yielded viable fresh hepatocytes was 249 ± 198 × 10^6^ cells per isolation, which equated to 4.36 ± 2.47 × 10^6^ cells per gram of perfused tissue. Hepatocyte viability was on average 84.1 ± 2.62% (Table 1) and is comparable to that reported by Bakala et al. (2003) of 82.7 ± 10.2% and Stefanski et al. (2013) of 84.6 ± 6.4%. However, the number of hepatocytes isolated per gram of perfused tissue was fivefold higher than that reported by Stefanski et al. (2013) (*n* = 9) but fivefold lower than in the study by Bakala et al. (2003). Although protocols were similar, Bakala et al. (2003) used a University of Wisconsin proprietary solution for transportation, which may have superior organ preservation capacities, enabling a larger viable cell yield to be obtained. Another potential factor is the number of liver samples, which was greater (*n* = 264) .

**Table 1:** Summary of equine liver cell viability and yield pre‐cryopreservation

	Age at slaughter (years)	Weight of liver (g)	Number of viable cells isolated per gram perfused tissue ×10^6^	Average cell viability (%)
1	19	100	4.10	87.7
2	18	90	9.33	83.0
3	3	100	3.83	81.0
4	6	90	4.88	83.7
5	18	80	1.6	82.6
6	14	80	2.4	87.7
7	25	80	4.4	82.7
Mean			4.36	84.1
SD			2.47	2.62

**Figure 1:**
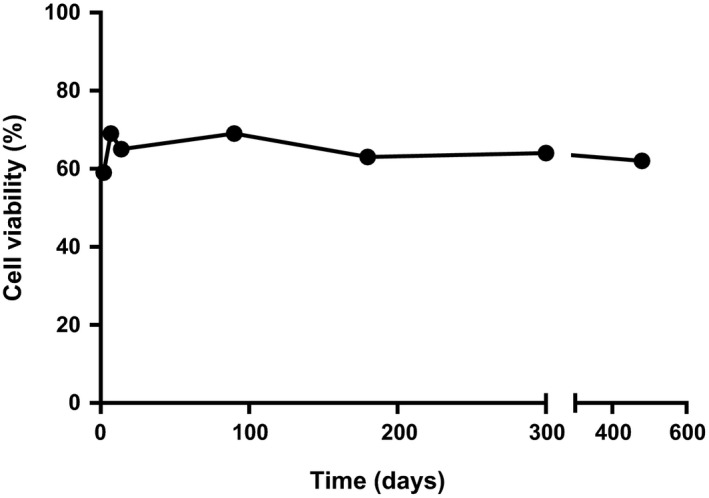
Effect of storage time on equine hepatocyte post‐thaw viability. Cryopreserved hepatocytes were stored in liquid nitrogen from 2 to 480 days. The results show that long storage of equine hepatocytes in liquid nitrogen has no effect on cell viability.

Cryopreserved equine hepatocytes had a post‐thaw viability of 60.3 ± 12% (Table 2). The results obtained in this study were consistent with data obtained from other species such as rats and human. For rat cryopreserved hepatocytes, the immediate post‐thaw viability of 60% was achieved by Sosef et al. (2005). Meanwhile, Terry et al. (2010) obtained post‐thaw viability of 52 ± 9% using cryopreserved human hepatocytes. A batch of cryopreserved equine hepatocytes were stored for up to 16 months in liquid nitrogen and no effect on post‐thaw viability was observed in that time frame (Fig. 1). Two concentrations of Percoll gradient (25% and 30%) were used in this study to examine their effect on the percentage viability and recovery. A 25% Percoll treatment increased hepatocyte viability from 52 ± 14 to 62.3 ± 11% with 31.6 ± 6% recovery (*n* = 3). However, the average viability after 30% Percoll treatment was 77 ± 11% with an average recovery of 27 ± 7% (Table 2). The recovery percentage was higher with 25% Percoll and is consistent with results reported by McGinnity et al. (2004). However, the post‐thaw viability after 25% Percoll reported herein was lower than the acceptable limit for enzyme kinetic studies. In their study, McGinnity et al. (2004), found that a 25% Percoll treatment increased dog and human hepatocyte viability from 52 ± 1 to 78 ± 8% and 66 ± 2 to 88 ± 3%, respectively. Therefore, a 30% Percoll concentration was used for kinetic studies since it gave a higher post‐thaw viability with an acceptable recovery. The Percoll density gradient has often been used for cell separation and to increase viability by separating live from dead cells (Kreamer et al. 1986; Innes et al. 1988; Diener et al. 1993). Moreover, several studies have found that the use of this gradient has an advantage for cell function, including cytochrome P450 function and cell attachment (Dou et al. 1992; Utesch et al. 1992; McGinnity et al. 2004).

**Table 2:** Post‐thaw viability with and without 30% Percoll gradient and number of viable cells after Percoll gradient

Horse	Recovered cryopreserved cells		
	Viability post‐cryo without Percoll gradient (%)	Viability post‐cryo with Percoll gradient (%)	Number of viable cells post‐cryo per vial ×10^6^
1	61.1	75.0	2.80
2	35.0	53.5	3.44
3	59.0	76.2	1.22
4	71.0	87.7	2.56
5	62.7	81.3	3.20
6	65.3	83.3	3.33
7	69.1	82.1	2.31
Mean	60.5	77.0	2.69
SD	12.0	11.0	0.8

The present work uses simple conventional commercially available solutions and tissue culture materials and methods for isolation and cryopreservation of equine hepatocytes with no need for specialized equipment or techniques and appears very effective. However, further optimization of the described methods may lead to an increased number of hepatocytes isolated per gram of perfused tissue and improvement of post‐thaw cell viability and function.

### Comparison of intrinsic clearance and metabolite formation between fresh and cryopreserved equine hepatocytes

**Figure 2:**
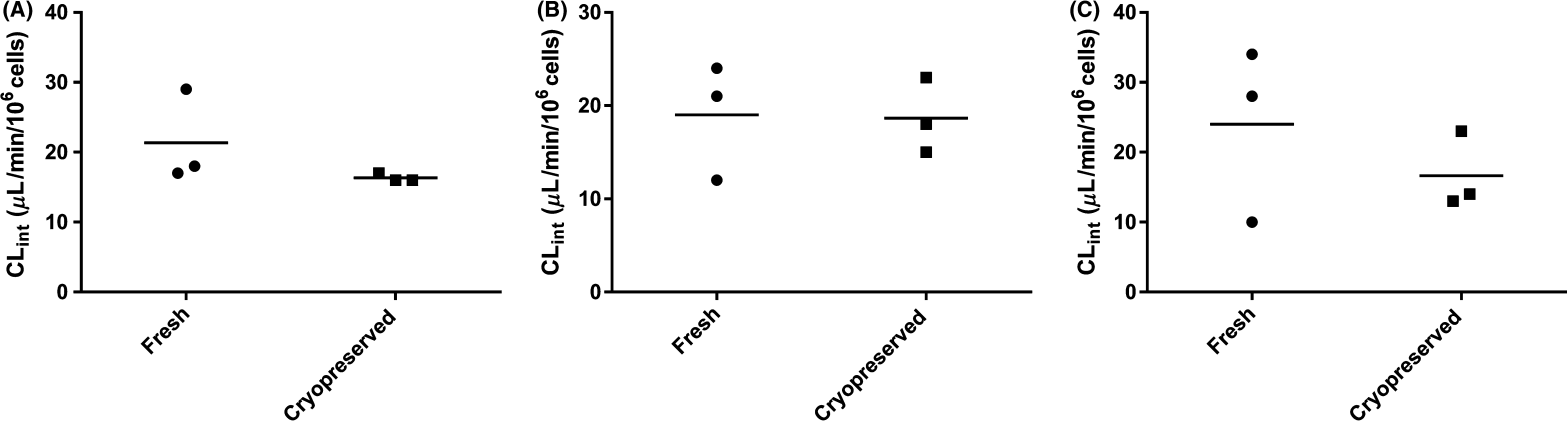
Comparison of intrinsic clearance between fresh and cryopreserved hepatocytes (mean presented by dash line) for omeprazole (A), flunixin (B), and phenylbutazone (C) (*n* = 3 horses for each drug). The results show that there is no significant difference between fresh and cryopreserved equine hepatocytes, *P* > 0.5.

This work also investigated the effect of cryopreservation on xenobiotic metabolizing enzymes. Intrinsic clearance is used as an indicator for the efficiency of metabolism and can be scaled to in vivo plasma clearance with knowledge of physiological parameters such as microsomal protein content, hepatocellularity, liver weight, and a model of hepatic extraction such as the well‐stirred model (Houston 1994; Naritomi et al. 2001). Intrinsic clearances for omeprazole, flunixin, and phenylbutazone were determined by the substrate depletion method. These drugs were chosen because they are known to produce metabolites from in vivo studies (Neto et al. 1996; Kanazawa et al. 2002; Jedziniak and Szprengier‐juszkiewicz 2005; Jedziniak et al. 2007). The results of this study show that there is no significant difference (*P* > 0.5) in intrinsic clearance between fresh and cryopreserved hepatocytes for the drugs investigated (Fig. 2). The mean ± SD (*n* = 3) of Cl_int_ for omeprazole, flunixin, and phenylbutazone were 21.3 ± 6.7, 16.3 ± 0.5, 19 ± 6.2, and 18.7 ± 4, 24 ± 12.49 and 16.7 ± 5.5 in fresh and cryopreserved hepatocytes, respectively.

**Figure 3:**
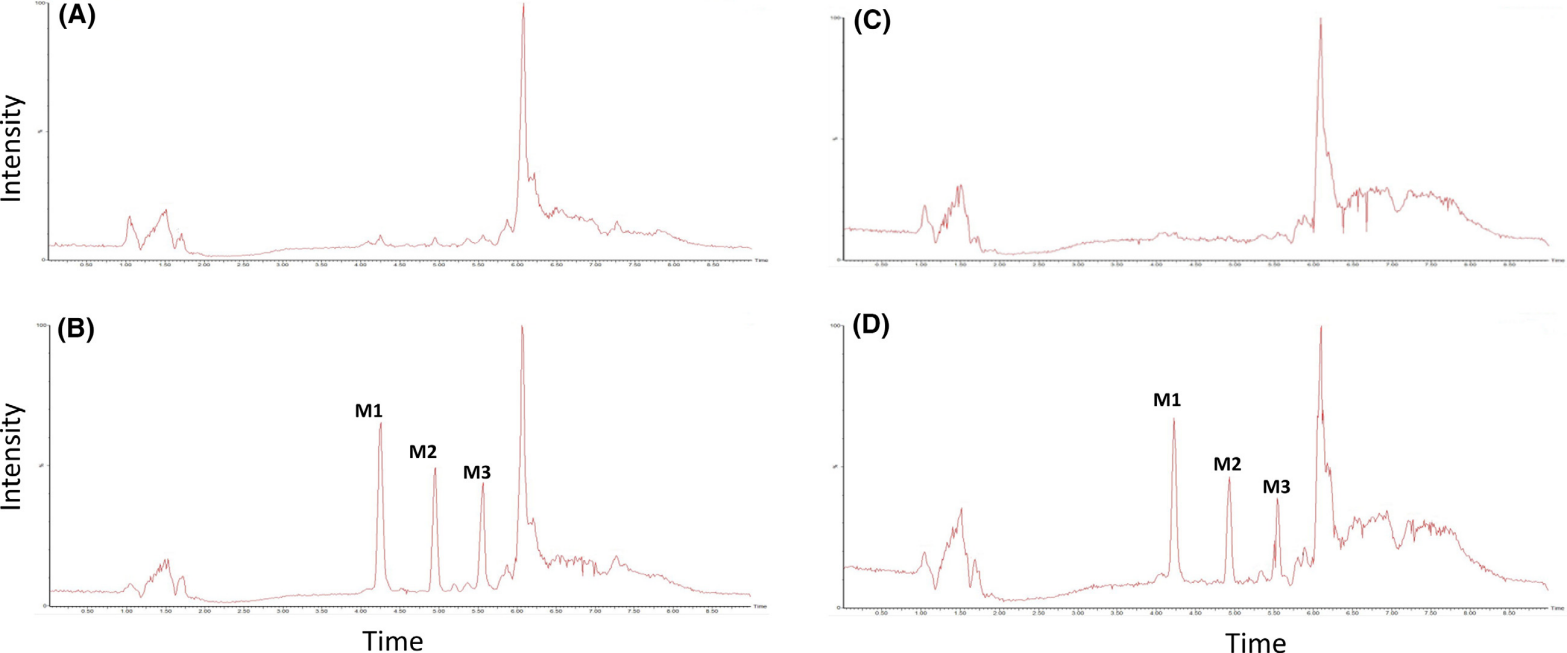
High‐performance liquid chromatography‐mass spectrometer (HPLC‐MS) chromatograms of oxidation metabolites of Omeprazole in suspension cultures of fresh (A = 0 min and B = 30 min) and recovered cryopreserved (C = 0 min and D = 30 min) equine hepatocytes. The ratio between the peak areas of M1, M2, and M3 was 2:1:1, respectively, in both fresh and cryopreserved hepatocytes.

**Figure 4:**
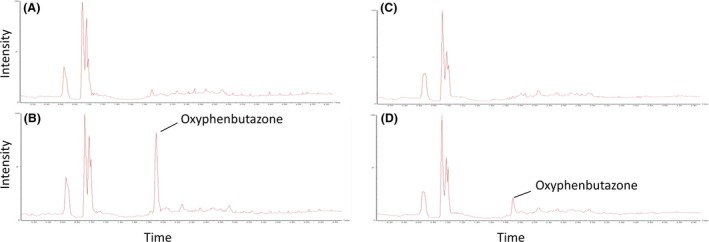
High‐performance liquid chromatography‐mass spectrometer (HPLC‐MS) chromatogram of oxidation metabolites of phenylbutazone in suspension cultures of fresh (A = 0 min and B = 30 min) and recovered cryopreserved (C = 0 min and D = 30 min) equine hepatocytes.

Moreover, Figure 3 shows the mass chromatograms of oxidation metabolites (MH+16/Z) generated for omeprazole in fresh (A and B) and cryopreserved (C and D) equine hepatocytes. Metabolites of omeprazole were characterized by comparison of the negative control chromatogram (0 time point, Fig. 3A and C) with the chromatogram of the 30 min time point (Fig. 3B and D). Oxidation metabolite peaks are presented by M1, M2, and M3 with retention times (RT) 4.22, 4.96, and 5.57 min. This suggests that M1 and M2 are more polar than omeprazole, whereas M3 is less. Kanazawa et al. (2002) determined a RT order of 5‐hydroxyomeprazole, omeprazole, and then omeprazole sulfone suggesting either M1 or M2 is 5‐hydroxyomeprazole and M3 is omeprazole sulfone. This study also identified 3‐hydroxyomeprazole as a minor metabolite, and although no RT has been published, it is a reasonable assumption that either M1 or M2 is 3‐hydroxyomeprazole. The ratio between peak areas of M1, M2, and M3 was 2:1:1, respectively, in both fresh and cryopreserved hepatocytes (Fig. 3A and B). A phenylbutazone oxidation metabolite was characterized by comparison of the negative control chromatogram (0 time point, Fig. 4A and C) with the chromatogram of the 30 min time point (Fig. 4B and D). This metabolite was identified as oxyphenbutazone since it showed an identical retention time to an authentic oxyphenbutazone standard. Oxyphenbutazone was detected for both fresh and cryopreserved hepatocytes, however, the intensity, after normalizing for internal standard, was 50% lower for the latter which may suggest some degradation in the metabolizing enzyme. No significant oxidation metabolites were observed for flunixin which is known to mainly undergo phase II metabolism in camel (Wasfi et al. 1998). Selected ion monitoring for glucuronide metabolites (MH+176/Z) showed no detectable metabolites. The observations observed in this study with equine hepatocytes are consistent with the literature for other species (Diener et al. 1993; McGinnity et al. 2004). Moreover, Griffin and Houston (2004) found that cryopreserved human hepatocytes gave similar high clearances for propranolol and diazepam compared to fresh hepatocytes.

In conclusion, this study has shown that equine hepatocytes can be successfully cryopreserved using a conventional freezing protocol. The use of a Percoll centrifugation after thawing yielded equine hepatocytes with high viability, which appear to possess an enzymatic metabolic capability similar to that of freshly isolated hepatocytes. A continuous availability of cryopreserved equine hepatocytes represents a major advantage as a tool for drug screening in the horse.

## Author Contributions

Participated in research design: K. A. Shibany, S. Tötemeyer, S. L. Pratt, S. W. Paine; Conducted experiments: K. A. Shibany and S. L. Pratt; Performed data analysis: K. A. Shibany and S. L. Pratt; Contributed to writing manuscript: K. A. Shibany, S. Tötemeyer, S. L. Pratt, S. W. Paine.

## Disclosures

None declared.
